# Switch from Cap- to Factorless IRES-Dependent 0 and +1 Frame Translation during Cellular Stress and Dicistrovirus Infection

**DOI:** 10.1371/journal.pone.0103601

**Published:** 2014-08-04

**Authors:** Qing S. Wang, Eric Jan

**Affiliations:** Department of Biochemistry and Molecular Biology, University of British Columbia, Vancouver, British Columbia, Canada; The John Curtin School of Medical Research, Australia

## Abstract

Internal ribosome entry sites (IRES) are utilized by a subset of cellular and viral mRNAs to initiate translation during cellular stress and virus infection when canonical cap-dependent translation is compromised. The intergenic region (IGR) IRES of the *Dicistroviridae* uses a streamlined mechanism in which it can directly recruit the ribosome in the absence of initiation factors and initiates translation using a non-AUG codon. A subset of IGR IRESs including that from the honey bee viruses can also direct translation of an overlapping +1 frame gene. In this study, we systematically examined cellular conditions that lead to IGR IRES-mediated 0 and +1 frame translation in *Drosophila* S2 cells. Towards this, a novel bicistronic reporter that exploits the 2A “stop-go” peptide was developed to allow the detection of IRES-mediated translation *in vivo*. Both 0 and +1 frame translation by the IGR IRES are stimulated under a number of cellular stresses and in S2 cells infected by cricket paralysis virus, demonstrating a switch from cap-dependent to IRES-dependent translation. The regulation of the IGR IRES mechanism ensures that both 0 frame viral structural proteins and +1 frame ORFx protein are optimally expressed during virus infection.

## Introduction

The majority of eukaryotic mRNAs utilize a cap-dependent scanning mechanism to recruit the ribosome, whereas internal ribosome entry sites are cis-acting elements that direct recruitment of the ribosome in a 5′ end independent manner. Initially discovered in picornaviruses, IRESs have been found in other viruses including flaviviruses, retroviruses, and dicistroviruses and in a subset of cellular mRNAs [1], [2]. IRESs, in general, adopt RNA structures that recruit specific translation initiation factors or IRES trans-acting factors (ITAFs) that contribute to ribosome recruitment and translation initiation [3], [4]. In general, within each viral family, the factor requirements for IRES translation are unique [5] which likely reflects the mechanism by which translation is inhibited during virus infection. For example in poliovirus infection, the viral protease targets not only the viral polyprotein but also translation initiation factors, eIF4G and PABP, resulting in the shutoff of host translation. In contrast, the polioviral IRES can still utilize the cleaved C-terminal fragment of eIF4G to mediate viral protein translation [6]. It is proposed that the IRES allows for preferential translation of an mRNA during cellular stress or viral infection when overall cap-dependent translation is compromised [1], [7]. Coordination of this switch from cap-dependent translation to IRES-dependent translation is an important strategy utilized by some positive strand RNA viruses to efficiently hijack the ribosome for productive viral protein synthesis [1], [7].

**Figure 1:**
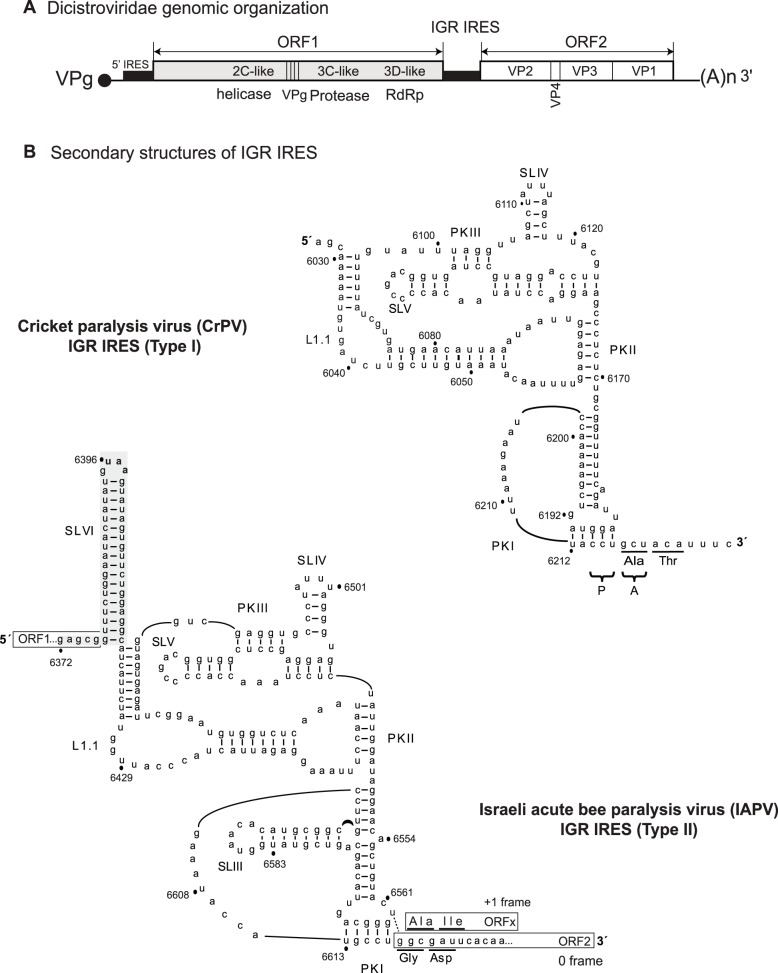
Secondary structures of the CrPV (Type I) and IAPV (Type II) IGR IRESs. (A) Distinct IRESs direct translation of nonstructural (ORF1) and structural (ORF2) polyproteins. (B) Schematic of the IRESs showing pseudoknots, PKI, PKII, and PKIII, stem loops SLIII, SLIV, SLV, and SLVI, and loop L1.1. The UAA stop codon of the IAPV ORF1 is shown in bold. The overlapping +1 frame ORFx is shown. Translation of IAPV ORFx is mediated by a U-G base pair adjacent to PKI (dashed lines).

Among them, the intergenic region IRES (IGR IRES) of the *Dicistroviridae* family utilizes one of the most unique mechanism to initiate translation. Dicistroviruses possess a single-strand positive sense RNA genome, which contains two open reading frames encoding the structural and nonstructural proteins, each of which is driven by a distinct IRES (Figure 1A) [8]–[10]. Members of the *Dicistroviridae* family infect arthropods including Drosophilidae, Aphidoidea, Caridea, and Apidae and include the cricket paralysis virus (CrPV), Drosophila C virus (DCV), and the honey bee viruses such as the Israeli acute paralysis virus (IAPV), acute bee paralysis virus (ABPV), and Kashmir bee virus (KBV) [8]. The intergenic region IRES (IGR IRES) of the *Dicistroviridae* family adopts a unique triple-pseudoknot RNA structure (Figure 1B) to direct ribosome recruitment without the need of initiation factors [11]–[17]. Moreover, structural and biochemical studies have revealed that the IRES functionally mimics a tRNA to hijack and manipulate the ribosome [17]–[19]. Pseudoknots II and III (PKII and PKIII) compose one domain which binds to 80S, whereas the tRNA-like PKI domain occupies the ribosomal P-site and directs translational initiation from a non-AUG codon in the ribosomal A-site (Figure 1B) [11], [12], [15], [17]–[20]. Recent cryo-EM structures of IGR IRES/ribosome complexes have provided additional insights into this mechanism: the PKI domain first occupies the ribosomal A site and translocation of the IRES by eEF2 occurs prior to delivery of the first aminoacyl-tRNA [21], [22].

The IGR IRESs can be classified into two types. Type I and II IGR IRESs are exemplified by the CrPV and IAPV IGR IRES, respectively (Figure 1B). Type II IRESs contain an extra stem loop (SLIII) within the PKI domain and a longer L1.1 region (Figure 1B). We previously showed that the PKI domains can be swapped between Type I and II IGR IRESs without compromising function [23]. Within the Type II IGR IRESs, the honey bee viruses also contain an extra 14–18 bp stem loop (SLVI), which enhances IGR IRES translation *in vitro* [24]–[26]. Recently, we demonstrated that a subset of Type II IGR IRESs that include the honey bee virus, IAPV, can direct translation in both the 0 and +1 frames to synthesize the viral structural proteins and a hidden +1 frame protein, called ORFx [25]. An U-G base pair (U6562/G6618) adjacent to the PKI domain is necessary to shift the reading frame of the ribosome into the +1 frame *in vitro* (Figure 1B) [25]. ORFx is expressed in virally-infected honey bees suggesting that its expression is important for viral infection [25]. However, the role of ORFx is currently not known. Furthermore, it has not been investigated whether +1 frame translation directed by the IGR IRES is regulated distinctly of 0 frame translation.

Infection by dicistroviruses leads to a shutoff of host protein synthesis concomitant with preferential viral translation [27]–[29]. To date, the mechanisms underlying the translational shutoff during dicistrovirus infection is not completely understood. Previously, we showed that eIF2α is phosphorylated and eIF4E-eIF4G interactions are compromised during infection [27]. Both mechanisms likely contribute to the inhibition of host translation, which in turn leads to preferential IRES-dependent translation of the viral nonstructural and structural proteins during infection [27]. Furthermore, the structural proteins are expressed in molar excess of the nonstructural proteins, suggesting that IGR IRES translation is stimulated during infection [27]–[29]. As IGR IRES exemplifies one of the more unusual mechanisms for translational initiation and for ribosome recruitment, an outstanding question is why dicistroviruses may have evolved this type of mechanism. A probable explanation may be due to, as discovered during poliovirus infection [6], [30], the availability of cellular and viral factors including many initiation factors during dicistroviruses infection. It is therefore predicted that IGR IRES, which can assemble ribosome without factors, would be translated under many situations when cap-dependent translation is compromised. However, other alternative explanations exist such as the possibility that the replicating viral RNA simply outcompetes the host mRNAs for the translational machinery. Here, we have systematically explored IGR IRES-dependent translation in insect cells under dicistrovirus infection as well as during cellular stresses that compromise different initiation factors. Toward this, we established a transfection approach using capped dicistronic reporter RNAs to monitor both cap- and IGR IRES-mediated translation. We also developed T2A-containing bisictronic constructs to maximize detection of reporter luciferase activities. Using these assays, we determined whether 0 and +1 frame IGR IRES-mediated translation are differentially regulated *in vivo*.

## Materials and Methods

### Bicistronic reporter constructs

Each IGR IRES with flanking upstream and downstream sequences was cloned between the *Eco*RI site and the *Nco*I site within the intergenic region of plasmid pEJ551[31], which was based on the bicistronic construct pRΔDEF first described by the Sarnow group [32]. The Firefly luciferarse (FLuc) gene was fused in frame to either the 0 or +1 frame. The sequences that were cloned are as follows: nucleotides 5974–6372 of Cricket paralysis virus (CrPV, accession: NC_003924.1), nucleotides 6372–6908 of Israel acute paralysis virus (IAPV, accession: NC_009025), nucleotides 6296–6814 of acute bee paralysis (ABPV, accession: NC_002548), nucleotides 6381–6908 of Kashmir bee virus (KBV, accession: NC_004807), and nucleotides 4189–4797 of Solenopsis invicta virus (SINV-1, accession: NC_006559). For all IAPV IRES-containing bicistronic constructs, the AUG start codon of the FLuc gene was removed by PCR-based site-directed mutagenesis (Stratagene). In the case of bee paralysis viruses including IAPV, ABPV and KBV, reporter constructs were generated such that the UAA stop codon within SLVI serves as the termination codon for the reporter gene Renilla luciferase (RLuc).

**Figure 2:**
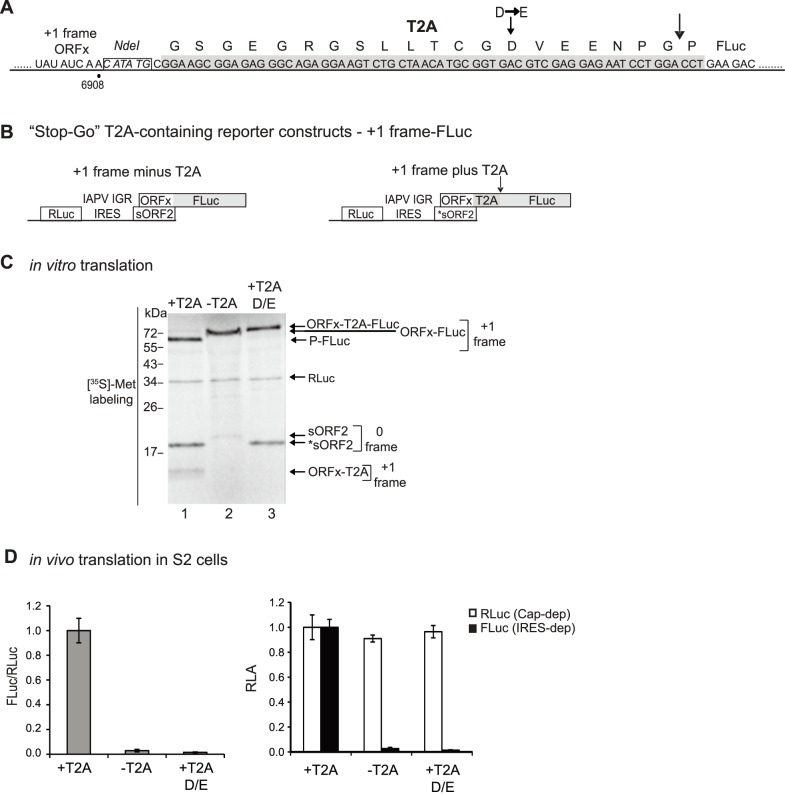
Construction of a T2A-containing +1 frame IRES bicistronic reporter construct. (A) The T2A sequence (dark grey) is inserted between an NdeI restriction site (boxed and italicized) and the Firefly luciferase (FLuc) gene. The arrow indicates the ‘self-cleavage’ or ‘stop-go’ site. A mutation within the T2A peptide (D to E), which inactivates T2A ‘self-cleavage’ activity is shown. (B) Bicistronic reporter constructs containing the IAPV IGR IRES and the ORFx region fused in the +1 frame with the FLuc gene. The T2A coding sequence (in grey) is inserted between the ORFx and FLuc. T2A-minus (left) and T2A-containing (right) bicistronic reporter constructs are shown. (C) T2A-minus and T2A-containing +1 frame bicistronic constructs were incubated in Sf21 extracts for 120 minutes in the presence of [^35^S]-methionine and analyzed by SDS-PAGE and autoradiography. (D) *In vivo* translation in S2 cells. *In vitro* transcribed 5′ capped bicistronic reporter RNAs were transfected into *Drosophila* S2 cells. Cells were harvested at 6 hours, lysed and luciferase activity was measured. The white and black boxes represent RLuc and FLuc luciferase expression, respectively, indicative of cap-dependent and IRES-dependent translation. Relative luciferase activities (RLA), the quantitation of FLuc and RLuc enzymatic activity, and the relative ratios of FLuc/RLuc are normalized to that observed with the +1 frame T2A-containing reporter RNA. Shown are averages from at least three independent experiments (± s.d.).

For the T2A-containing constructs, the *Thosea asigna* virus (accession: AF062037) 2A sequence (Figure 2A) was inserted in frame and preceding the FLuc gene by using an overlapping PCR strategy as described previously [31].

### Transfection of RNA into S2 cells

Capped reporter RNAs were obtained by *in vitro* transcription in the presence of cap analog [m^7^G(5′)ppp(5′)G] (Ambion) to GTP at a 5∶1 ratio as previously described [31]. RNAs were purified by RNeasy (Qiagen), their integrity confirmed by denaturing agarose gel analysis, and RNA concentration determined using a Nanodrop ND-1000 spectrophotometer.

*Drosophila* S2 cells were grown and passaged in M3+BPYE medium plus 10% FBS at 25°C. For transfection, 3×10^6^ S2 cells were plated per well for 1 hour to let the cells adhere to the bottom of the six well plate. The growth media was replaced with 2 ml fresh media and incubated for 3 hours. Capped bicistronic reporter RNAs (2 µg) were transfected into S2 cells with 5 µl of Lipofectamine 2000 (Invitrogen) in a total volume of 500 µl of reduced serum medium (Invitrogen, OPT-MEM I) following manufacture’s protocol.

Where indicated, 4 mM of Dithiothreitol (DTT), 8 µM of 4E1RCat (TOCRIS Bioscience) or a range of pateamine A (PatA) from 12.5 to 200 nM (generous gift of Jerry Pelletier, McGill University) were added to cells 1 hour after transfection. For virus infection experiments, S2 cells were infected on plates with CrPV at an MOI of 25 in 200 µl of PBS with constant rocking at 25°C. After 30 minutes of infection, 2 ml of conditional media was added back to each well, which was immediately followed by the addition of capped reporter RNAs mixed with Lipofectamine 2000 in reduced serum medium.

Unless indicated, cells were harvested 6 hour after transfection by centrifugation at 9600 g for 1 minute, followed by wash with 1 ml of PBS. The cells pellets were then resuspended in 40 µl of Passive Lysis Buffer (Promega) and stored at –20°C. The concentration of protein in each lysate was determined by Bradford assay.

### *In vitro* translation assay

In a 10 µl reaction, either 1 µg linearized bicistronic reporter constructs or 500 ng of capped reporter RNAs were incubated with 6.7 µl of Sf21 cell extract (Promega), 0.3 µl L- [^35^S]-methionine (PerkinElmer, >1000 Ci/mmol), 40 mM KOAc and 0.5 mM MgCl_2_ at 30°C for 1.5–2 hours as indicated [31]. Capped RNAs were preheated at 65°C for 3 minutes and incubated in buffer E (20 mM Tris pH 7.5, 100 mM KCl, 2.5 mM MgOAc, 0.25 mM Spermidine, and 2 mM DTT) at room temperature for 10 min before adding to the reaction. Proteins were separated by a 16% SDS polyacrylamide gel, exposed to a phosphor storage screen (Molecular Dynamic) and quantified using a Typhoon imager 8600 (Amersham). When calculating the ratio of FLuc/RLuc by [^35^S]-methionine labeling, the number of methionines were taken into account of each reporter protein.

### Dual-Luciferase Reporter Assay

A 96-well plate was plated with equal amounts of protein (∼100 µg total protein from S2 cells or 1 µl of *in vitro* translation reaction). 100 µl of Luciferase Assay Reagent II (Promega, Dual luciferase assay) was dispensed to determine FLuc activity using 10 second time intervals. Subsequently, 100 µl of Stop & Glo reagent (Promega, Dual luciferase assay kit) was dispensed to measure RLuc activity with the same time interval. A microplate luminometer (Berthold Technologies, Centro LB 960) supplied with software MikroWin 2000 was used to detect the luminescence signal, which is then normalized to obtain the relative luciferase activity (RLA). The quantitation of RLuc and FLuc activities were calculated separately and as a ratio of FLuc/RLuc.

## Results

### Establishment of a T2A-containing bicistronic construct to monitor IRES translation

In order to monitor IGR IRES-mediated translation *in vivo*, we cloned the IAPV and CrPV IRES within the intergenic region of the previously described bicistronic reporter construct [31]. We also created constructs where specific 0 and +1 frame translation mediated by the IAPV IGR IRES can be monitored. We briefly describe the construction of the reporters that were used in this study.

For all constructs, expression of FLuc is IRES-dependent and RLuc is cap-dependent. Sequences upstream and downstream of the IRES were also included as we and others have shown that inclusion of these sequences enhances IRES translation [19], [25], [31], [33]. Specifically, the downstream region of the CrPV (nucleotides 6217–6372) and IAPV ORF2 (called short ORF2 or sORF2, nucleotides 6618–6908), which includes the overlapping IAPV ORFx gene, were cloned in frame with the FLuc gene (Figure S1A, S1B). The starting codons of the IAPV and CrPV structural proteins are glycine GGC and alanine GCU codons, respectively. To monitor IAPV IGR IRES-mediated +1 frame translation, we created mutations within the ORFx sequence such that ORFx is fused in frame with FLuc (Figure 2B). In generating these constructs, a concern was that inclusion of viral protein sequences in frame with the reporter FLuc ORF may affect the luciferase enzymatic activity, thereby underestimating the actual IRES activity [31]. To circumvent these issues, we developed a novel bicistronic reporter construct which exploits the properties of the 2A ‘self-cleaving’ peptide (Figure 2A) [31].The 2A peptide stalls translating ribosome by interacting with the exit channel leading to codon-independent translational termination [34]. Ribosomal pausing results in nascent peptide cleavage between the two final amino acids of the peptide, glycine and proline. However, instead of ribosome dissociating from the mRNA, the ribosome continues translation starting from the proline codon. The 2A peptide, termed ‘stop-go’ or ‘stop-carry’ translational recoding mechanism, is used by several positive strand RNA viruses to produce separate proteins during translation of the polyprotein. For our studies, we chose the T2A peptide from the insect virus, *Thosea asigna*, which has been shown to be one of the most efficient and highly active in insect cells [35]–[37]. We subcloned the T2A peptide directly upstream and in frame of the FLuc ORF, thereby creating T2A-constructs that monitor both IAPV IGR IRES-mediated 0 (Figure S1, B) and +1 frame (Figure 2B) translation.

We first tested whether there were differences in IGR IRES-mediated 0 frame translation between T2A-less and T2A-containing bicistronic reporter constructs (Figure S1). We previously showed that IGR IRES translation can be assayed by incubating bicistronic reporter constructs in a Sf21 translation extract system and monitoring protein synthesis by either incorporation of radioactive [^35^S]-methionine or quantitation of luciferase enzymatic activities [25], [31]. As shown previously, a bicistronic reporter construct (minus T2A) containing the IAPV IGR IRES resulted in expression of three radiolabelled proteins, RLuc (scanning-dependent) and IGR IRES-dependent 0 frame sORF-FLuc and +1 frame ORFx (Figure S1, C lane 2) [31]. Mutations that disrupt PKI basepairing (ΔPKI, CC6615-6GG) (Figure S1, A) abolished expression of 0 and +1 frame proteins, indicating that IRES translation was being measured (Figure S1, C lane 1). In the case of the T2A-containing bicistronic construct (plus T2A), the T2A ‘self-cleaving’ activity led to the expression of two 0 frame translation products, sORF2-T2A and the FLuc protein with a proline at the N-terminus (proline-FLuc, P-FLuc) (Figure S1, C lane 3). In addition, because sequences downstream of the IRES were included, +1 frame ORFx protein was also synthesized (Figure S1, C lane 3). The mutant ΔPKI IRES did not result in expression of either 0 or +1 frame translation products, again confirming that IRES translation was being assayed (Figure S1, C lane 4). The ratio of FLuc/RLuc was measured either by quantitiating [^35^S]-methionine incorporation or by measuring the enzymatic luciferase activity (Figure S1, D and E). IGR IRES-mediated 0 frame FLuc enzymatic activity was not significantly affected in the presence or absence of the T2A peptide (Figure S1, E). Thus, for most of the studies, 0 frame translation directed by the IGR IRES was monitored using the T2A-less reporter constructs. To confirm that IRES translation was monitored using the coupled transcription-translation Sf21 system, we incubated *in vitro* transcribed 5′capped reporter RNA (Figure S1, F) in the Sf21 translation extract system. Capped reporter RNAs were obtained by using *in vitro* transcription reactions in the presence of cap analog m^7^G(5′)ppp(5′)G [31]. As expected, reporter RNAs containing the mutant ΔPKI IRES did not result in expression of the FLuc activity.

In contrast, FLuc enzymatic activity monitoring IAPV IGR IRES-mediated +1 frame translation was significantly affected in the presence of T2A [31]. Note that the T2A-containing +1 frame reporter constructs resulted in the synthesis of RLuc, the +1 frame cleaved proline-FLuc (P-FLuc) and ORFx-T2A proteins and the 0 frame *sORF2 (Figure 2C, lane 1). Calculation of the amount of P-Luc to the full-length ORFx-T2A-FLuc indicated >95% T2A-mediated ‘self-cleavage’ activity (Figure 2C). By comparison of the [^35^S]-methionine incorporation and the FLuc enzymatic activity in the Sf21 translation extracts, we have shown previously that the fusion of ORFx in-frame with the FLuc ORF inhibits FLuc enzymatic activity and that the inclusion of a functional T2A peptide into the bicistronic reporter system rescues the sensitivity of luciferase assay (Figure 2C) [31]. Inclusion of a mutant T2A peptide (D/E), which inactivates the ‘self-cleaving’ activity resulted in the full-length +1 frame ORFx-T2A-FLuc protein (Figure 2C, lane 3) [31].

To test whether the +1 frame T2A containing construct can rescue luciferase enzyme sensitivity *in vivo*, *in vitro* transcribed capped bicistronic RNAs were transfected into *Drosophila* S2 cells. We previously reported that the luciferase activities of an IRES-containing bicistronic reporter RNA increased linearly up to 6 hours post transfection and the maximal luciferase activity occurred 6–10 hours post transfection [31]. These findings argue that the reporter RNA is intact and engaged in translation during the first 6 hours after transfection. Therefore, cells were collected and FLuc activities were detected at 6 hours post transfection (Figure 2D). As shown *in vitro* [31], the IRES-dependent +1 frame FLuc luciferase signal was detected in S2 cells after transfection with a functional T2A-containing reporter RNA and not with a T2A-minus or mutant T2A (D/E) reporter RNA (Figure 2D). For the rest of these studies, we have used the T2A-containing +1 frame reporter constructs to monitor +1 frame translation *in vivo*.

### IGR IRES 0 frame translation in *Drosophila* cells

**Figure 3:**
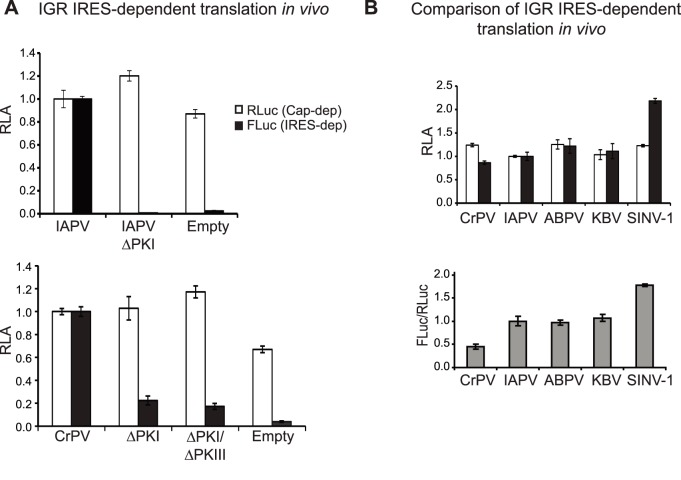
IRES-mediated translation in *Drosophila* S2 cells. (A) *In vitro* transcribed 5′ capped bicistronic RNAs containing wild-type or mutant CrPV and IAPV IGR IRESs were transfected into *Drosophila* S2 cells. Cells are harvested at 6 hours after transfection, lysed and luciferase activity was measured. To confirm CrPV IGR IRES dependent activity, reporter RNA containing a CrPV double mutant (ΔPKI/ΔPKIII) bearing both ΔPKI (CC6214-5GG) and ΔPKIII (CAC6148-50GUG) mutations and an empty construct, which denotes a bicistronic RNA that does not have an IRES were assayed. FLuc, RLuc, and the ratio of FLuc/RLuc are normalized to that observed with reporter RNAs containing the CrPV IGR IRES and IAPV IGR IRES, respectively. (B) Comparison of different dicistrovirus IGR IRES translation in S2 cells. FLuc, RLuc, and the ratio of FLuc/RLuc are normalized to translation of the reporter RNA containing the IAPV IGR IRES. Shown are averages from at least three independent experiments (± s.d.).

0 frame FLuc activity mediated by the IAPV [31] and CrPV IGR IRESs was compared to an empty bicistronic reporter construct (Figure 3A). As expected, mutations that inhibit IGR IRES translation, such as disrupting PKI basepairing (ΔPKI) (Figure S1, A) or both PKI and PKIII basepairing (ΔPKI/ΔPKIII (CAC6148-50GUG) in CrPV IGR IRES) resulted in significantly lower FLuc activity (Figure 3A). In *in vitro* translation experiments, these mutant IRESs are inactive [12], however, in Drosophila cells using our transfection protocol, we observed ∼20% residual translation (Figure 3A). Investigations into this phenomenon are ongoing but it is possible that these mutant IRESs may still adopt a core structure that can still drive residual IRES translation *in vivo*. It is most probable that the bulk luciferase activity detected is IRES-dependent. It has been proposed that the adjacent sequence downstream of IGR IRES is unstructured to allow the IRES to adopt its conformation for optimal translation [33]. To confirm whether sequences adjacent to the CrPV IGR IRES can affect IRES activity as shown in IAPV IGR IRES [25], [31], we compared translational activities using a reporter RNA containing either the minimal core CrPV IGR IRES (nucleotides 6025–6231) or the IRES with sequences adjacent to the core IRES (nucleotides 5974–6372) (Figure S2, A). The FLuc enzymatic activity was not affected when the FLuc ORF was fused with the sequences adjacent to the core IRES (Figure S2, A). The CrPV IRES containing regions adjacent to the core IRES was 5 fold more active than the minimal core IRES *in vivo* and ∼2 fold *in vitro* (Figure S2, A and B).

To ensure that cap-dependent translation was being measured, we compared the expression of RLuc activity of cells transfected with either 5′ capped and uncapped bicistronic RNAs. The 5′ capped RNA significantly displayed more RLuc activity than uncapped RNA (∼20 fold), indicating that cap-dependent translation is being assayed in this transfection approach (Figure S2, C).

We next compared translation from a panel of dicistrovirus IGR IRESs including the honey bee viruses ABPV and KBV and the fire ant SINV-1 virus. Similar to the cloning strategy for the IAPV and CrPV IGR IRESs, we subcloned sequences adjacent to the IRES to ensure optimal translational activity (see Materials and Methods for details). In S2 cells, the fire ant dicistrovirus SINV-1 IGR IRES showed the highest translational activity, approximately 2-fold higher than that from the IAPV, ABPV and KBV IGR IRESs. As a comparison, the CrPV IGR IRES had the lowest translational activity under basal conditions (Figure 3B). We also found that IAPV IGR IRES-dependent translation can be readily detected in another *Drosophila* cell line, Kc167 cells (Figure S3, A). In summary, we have established a general protocol to monitor IGR IRES-mediated translation in *Drosophila* cells.

### IAPV IGR IRES +1 frame translation in *Drosophila* S2 cells

**Figure 4:**
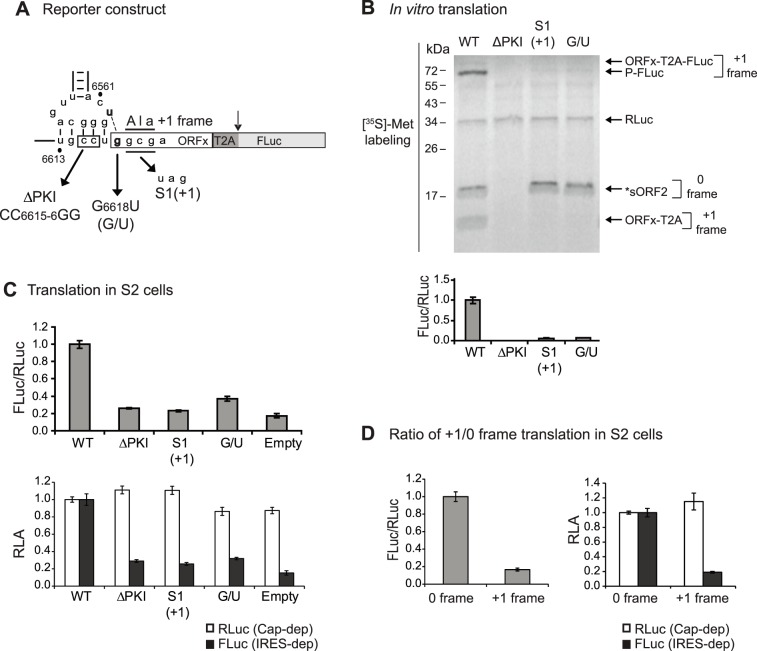
IAPV IRES-mediated +1 frame translation using the T2A-containing reporter construct. (A) Schematic of mutations used within the PKI domain of the IAPV IGR IRES. (B) Translational activity of reporter constructs containing wild-type or mutant IAPV IGR IRESs in Sf21 extracts. Bicistronic reporter constructs were incubated in Sf21 extracts in the presence of [^35^S]-methionine. (below) Quantitation of the FLuc/RLuc ratio (below) is normalized to the wild type ratio. (C) Translational activity in *Drosophila* S2 cells. *In vitro* transcribed capped T2A-containing reporter RNAs were transfected into S2 cells. Cells were harvested at 6 hours after transfection and luciferase activities were measured. (D) Comparison of 0 and +1 frame IAPV IGR IRES-mediated translation of T2A-containing reporter RNAs transfected in S2 cells. Luciferase activities were quantitated 6 hours after transfection. For C) and D), luciferase activities are shown as a ratio of FLuc/RLuc and as individual FLuc and RLuc activities. Shown are averages from at least three independent experiments (± s.d.).

We next investigated whether +1 frame translation directed by the IAPV IGR IRES can be monitored using the T2A-containing reporter construct in *Drosophila* S2 cells. Compared to a reporter construct which does not contain an IRES (empty) or a construct containing a mutant IRES (ΔPKI), +1 frame translation of FLuc using the wild-type IAPV IGR IRES-dependent FLuc translation was detected above background (Figure 4A, 4C). Previously, we showed that mutation of G6618 to U, which disrupts the U6562/G6618 base pairing adjacent to PKI, abolishes +1 frame ORFx translation [25]. The mutant G6618U IRES (G/U) resulted in loss of +1 frame FLuc translation both *in vitro* and in S2 cells to a level similar to the empty reporter construct (Figure 4A, 4B, lane 4 and 4C). A similar result was observed using the reporter construct with a stop codon in place of the +1 frame GCG starting codon (Figure 4A, 4B, lane 3 and 4C). Note that insertion of the stop codon in the +1 frame did not affect 0 frame *sORF2 expression (Figure 4B, lane 3), confirming that +1 frame translation was measured (Figure 4B, 4C). Thus, we report a T2A-containing bicistronic reporter construct that faithfully monitors IAPV IGR IRES-dependent +1 frame translation *in vivo*.

Using the *in vitro* translation assay, the ratio of +1/0 frame translation is calculated to be approximately 20% by [^35^S] methionine labeling [25]. For *in vivo* studies, we compared the FLuc/RLuc ratio from the transfection experiments in *Drosophila* S2 cells using reporter RNAs that monitor either +1 or 0 frame translation. In order to compare directly, we used IRES-mediated reporter RNAs that contained the T2A peptide. In transfected S2 cells, we found that the +1 to 0 frame translation ratio is approximately 20% (Figure 4D), which recapitulates the results observed *in vitro* [25].

### IGR IRES-mediated 0 and +1 frame translation during cellular stress

We next investigated whether 0 and +1 frame translation directed by the IAPV IGR IRES are differentially regulated. It has been established that the IGR IRES can direct 0 frame translation in mammalian and yeast cells and can be stimulated under cellular stress conditions when cap-dependent translation is compromised [17], [38]–[43]. To examine the extent to which the IGR IRES can direct 0 and +1 frame translation in S2 cells, we treated cells with different stressors, including DTT, pateamine A (PatA), or 4E1Rcat, each of which targets a specific step or activity in cap-dependent translation. DTT treatment induces ER stress and effectively leads to eIF2α phosphorylation and a shutdown of overall translation [27], [44]. PatA modulates eIF4A activity, thereby disrupting RNA helicase activity during cap-dependent translation [45]. 4E1Rcat binds to eIF4E and prevents the formation of the cap-binding complex [46]. The use of these compounds allows for systematic examinations into the cellular conditions that lead to IGR IRES translation.

**Figure 5:**
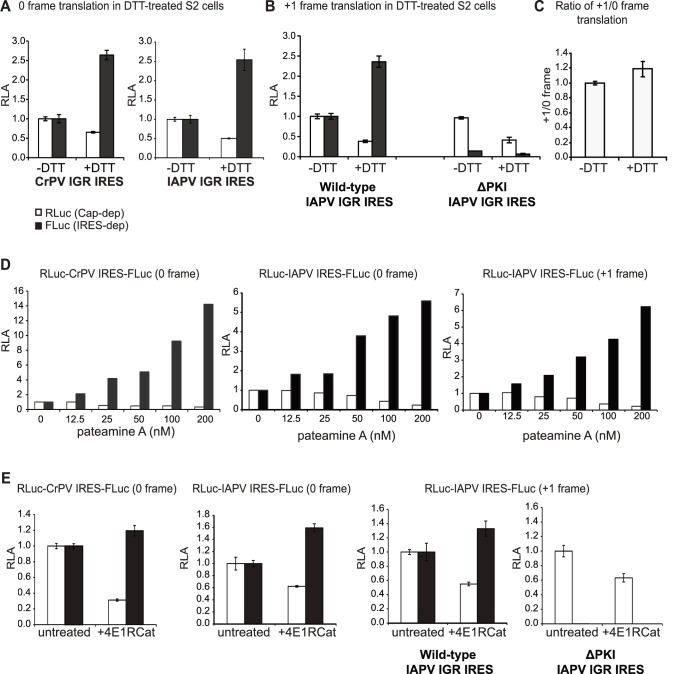
IGR IRES-mediated translation during cellular stress. CrPV and IAPV IGR IRES-mediated 0 and +1 frame translation was monitored in S2 cells treated with DTT (4 mM) (A–C), pateamine A (D), or 4E1RCat (8 µM) (E). Bicistronic reporter RNAs containing the CrPV or IAPV IGR IRES were transfected in S2 cells. After one hour transfection, cells were treated alone or with the drug for another 5 hours. (C) Relative ratio of IAPV IGR-mediated 0 and +1 frame translation of T2A-containing reporter RNAs transfected in DTT-treated S2 cells. Except in (D), shown are averages from at least three independent experiments (± s.d.).

IRES translation was monitored by transfection of 5′ capped bicistronic reporter RNAs in S2 cells treated with each stressor. Stressors were added to the cells at 1 hour after transfection and cells were harvested 5 hours later. As predicted, cap-dependent RLuc expression was inhibited by approximately 50% under DTT treatment (Figure 5A). In contrast, CrPV and IAPV IGR IRES-mediated 0 frame translation were stimulated under DTT treatment (Figure 5A). Specifically, both IRESs stimulated 0 frame translation by approximately 2.5 fold (Figure 5A). Similar to that observed with the 0 frame translation, +1 frame translation increased to the same extent during DTT treatment (Figure 5B). The ΔPKI mutant IRES did not display significant FLuc expression under basal or DTT treatment, again confirming that FLuc expression is IGR IRES-dependent (Figure 5B). We found that the relative 0 and +1 frame translation did not appear to change under basal or DTT treatment (Figure 5C). These results suggest that +1 and 0 frame translation by the IGR IRES is regulated similarly during DTT treatment.

A similar result is observed with cells treated with PatA and 4E1Rcat. Cap-dependent RLuc translation was significantly inhibited with increasing amounts of PatA treatment (Figure 5D). At 200 nM PatA, cap-dependent translation was inhibited by 60–80% (Figure 5D). In contrast, CrPV and IAPV IGR IRES-dependent 0 frame FLuc translation was stimulated under PatA treatments but to different extents. IAPV IGR IRES translation increased by approximately 2 to 6 fold under increasing concentrations of PatA (Figure 5D) whereas CrPV IGR IRES translation was stimulated 12 fold at the highest concentration of PatA (Figure 5D). Similarly, IAPV IGR IRES +1 frame translation was stimulated to the same extent during PatA treatment as 0 frame translation (Figure 5D).

4E1Rcat at 8 µM had a moderate inhibitory effect on cap-dependent translation by only 40–70% (Figure 5E). At higher 4E1RCat concentrations, both cap-dependent and IRES-dependent translation was significantly inhibited (data not shown). In contrast, 4E1RCat treatment at 8 µM stimulated CrPV and IAPV IGR IRES 0 frame translation by only approximately 1.2–1.5 fold (Figure 5E). 4E1RCat treatment also stimulated IAPV IGR IRES +1 frame translation by ∼1.3 fold whereas this treatment did not stimulate +1 frame translation with the IAPV IGR IRES ΔPKI mutant construct (Figure 5E). In summary, these results showed that IGR IRES-mediated 0 and +1 frame translation are stimulated in S2 cells to the same extent under different cellular stress conditions when overall translation is compromised.

### IGR IRES-mediated 0 and +1 frame translation during CrPV infection

**Figure 6:**
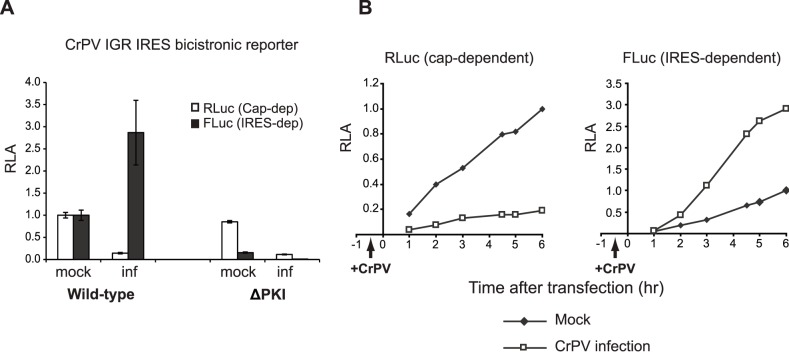
CrPV IGR IRES-mediated translation in CrPV-infected S2 cells. Bicistronic reporter RNAs containing the wild-type or mutant (ΔPKI) CrPV IGR IRES were transfected into mock or CrPV-infected (inf) S2 cells (MOI 25) at 0.5 hour post infection. (A) Cells were harvested at 6 hours after transfection (shown are averages from at least three independent experiments ± s.d.) or (B) at the indicated times and luciferase activities were measured. The RLuc and FLuc activities are normalized to that at 6 hours after transfection in mock-infected cells.

We next examined whether 0 and +1 frame IAPV IGR IRES-mediated translation is regulated differentially during dicistrovirus infection. The ideal experiment would be to monitor IAPV IGR IRES translation during honey bee dicistrovirus infection, however, to date, an appropriate honey bee cell line which can be infected with a pure honey bee dicistrovirus has not been established. As a result, we used S2 cells infected with a related dicistrovirus member, CrPV, as a model system. IRES-containing bicistronic reporter RNAs were transfected into S2 cells 30 minutes after CrPV infection and then harvested at either 6 hours or at the different time points post transfection. CrPV infection in S2 cells results in a rapid shutdown of host protein synthesis [27]. Similar to these findings, comparing to mock-infected cells, cap-dependent RLuc translation was significantly down regulated by 80–90% as early as 1.5–2.5 hours of infection and by 6.5 hours post infection (Figure 6A,6B). Note in Figure 6B, the RLuc and FLuc activities are normalized to that at 6 hours after transfection in mock-infected cells. In contrast, FLuc expression driven by the CrPV IGR IRES was significantly enhanced at all times post infection. At 6.5 hours post infection, CrPV IGR IRES-mediated translation was stimulated approximately 2.5–3 fold as compared to that during mock-infection (Figure 6A). As expected, the mutant ΔPKI IRES did not result in significant FLuc expression in mock- or CrPV-infected S2 cells (Figure 6A). We next examined the relative CrPV IGR IRES (FLuc) to cap-dependent (RLuc) translation ratio in mock- and CrPV-infected cells. In mock-infected cells, CrPV IGR IRES translation is 0.8% of cap-dependent translation, indicating that IRES translation is weak *in vivo*. Under virus infection, CrPV IGR IRES translation was 16% of cap-dependent translation, which is in part due to the 80–90% shutoff of overall cap-dependent translation and the stimulation of IRES translation. These results demonstrate that there is a switch from cap-dependent to IRES-dependent translation during CrPV infection.

**Figure 7:**
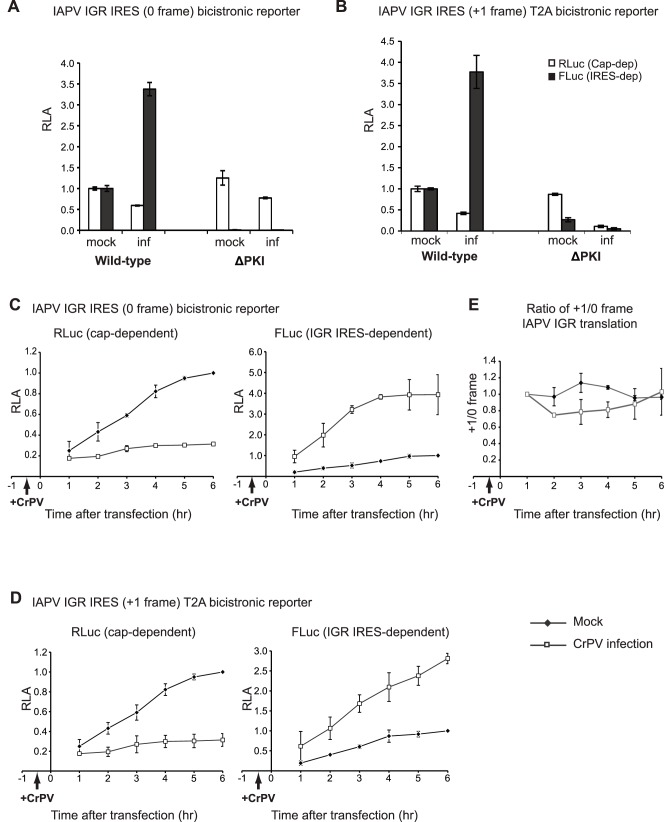
IAPV IGR IRES-mediated 0 and +1 frame translation in CrPV-infected S2 cells. (A, B) Bicistronic reporter RNAs containing the wild-type or mutant (ΔPKI) IAPV IGR IRES were transfected into mock or CrPV-infected (inf) S2 cells (MOI 25) at 0.5 hour post infection. (A, B) Cells were harvested at 6 hours post transfection or (C, D) at the indicated times and luciferase activities were measured. Reporter RNAs monitoring (C) 0 frame or (D) +1 frame translation are shown. The RLuc and FLuc activities are normalized to that at 6 hours after transfection in mock-infected cells. (E) Quantitation of the relative ratio of +1/0 frame IAPV IGR IRES translation in mock- or CrPV-infected cells. The ratios are normalized to that of the 1 hour after transfection in mock cells. Shown are averages from at least three independent experiments ± s.d.

Similar to that observed with the CrPV IGR IRES, transfection of reporter RNAs containing the IAPV IGR IRES resulted in stimulation of FLuc translation in both the 0 and +1 frames during CrPV infection (Figure 7). In CrPV-infected cells, both 0 and +1 frame IAPV IGR IRES translation was stimulated 2.5–5 fold as compared to that in mock-infected cells, whereas the cap-dependent RLuc expression was inhibited (Figure 7A–7D). The T2A-containing 0 frame reporter RNA showed a similar fold increase at 6.5 h.p.i. CrPV infection as that observed with the T2A-minus 0 frame reporter RNA (data not shown). As expected, mutations that disrupt PKI basepairing in the IAPV IRES abolished 0 and +1 frame FLuc expression in mock- and CrPV-infected cells (Figure 7A–7B). A time course following the expression of RLuc and FLuc showed that cap-dependent translation was inhibited early in infection and remained shutoff (∼90% inhibition) throughout infection. In contrast, IGR IRES-dependent 0 and +1 frame translation increased throughout the course of infection (Figure 7C–7D). Over the course of infection, the relative +1 to 0 frame translation did not appear to change significantly, suggesting that both 0 and +1 frame IAPV IRES-mediated translation were similar during CrPV infection (Figure 7E). We do note that at 3.5–4.5 h.p.i., the relative ratio of +1 to 0 frame translation decreases slightly, suggesting that 0 and +1 frame translation may be differentially regulated albeit to a minor extent. Similar to that observed with CrPV IRES translation, 0 frame IAPV IGR IRES translation was 1.1% and 11% of cap-dependent translation in mock- and CrPV-infected cells, indicating a switch from cap-dependent to IRES-dependent translation. In summary, these results demonstrate that IAPV IGR IRES translation is stimulated during CrPV infection.

## Discussion

Viruses are exquisitely dependent on host translational machinery, however, the strategies by how this is achieved are diverse [7], [47]. For some RNA viruses such as picornaviruses, specific steps in cap-dependent translation are compromised thus increasing the available pool of ribosomes and initiations factors for viral IRES translation. In contrast, DNA viruses like human cytomegalovirus (HCMV), host translation is not impaired and virus replication relies on stimulation of cap-dependent translation machinery and increases in initiation factors concentration [48], [49]. Other viruses target host mRNA metabolism. For example, vesicular stomatitis virus inhibits the nuclear export of cellular mRNAs [50], and severe acute respiratory virus (SARS) suppresses host translation by inactivating 40S ribosomes and selectively promoting host but not viral RNA degradation [51], [52]. In the case of herpes virus infection, host translation is inhibited in part through degradation of host mRNAs, whereas the accelerated viral mRNA turnover helps regulate different population of viral mRNAs expression. [53]–[55]. Alternatively, it has been proposed that replicating viral RNA genomes can outcompete for host factors and ribosomes for viral protein synthesis.

Infection by dicistroviruses leads to a rapid inhibition of host translation concomitant and a switch to viral translation [27], [28]. To mimic these conditions, we treated cells with DTT, PatA, and 4E1RCat, each targeting specific initiation factors. In all cases, IRES dependent translation is stimulated under these conditions in Drosophila cells (Figure 5). Although we cannot completely rule out that stimulation of IRES-mediated translation is due induction of apoptosis at later time points of drug treatment, it is notable that these results are similar to that observed in mammalian and yeast cells yeast cells [17], [38]–[41], [43], [46] and are consistent with the property that the IGR IRES does not require factors for ribosome assembly. Using our transfection protocol of reporter bicistronic RNAs, we also find that both CrPV and IAPV IGR IRESs are stimulated during CrPV infection (Figure 6–7). These results argue that the stimulation of IGR IRES-mediated translation during infection is not due to the replication of viral RNAs simply outcompeting host mRNAs for ribosomes. To our knowledge, this is the first report addressing IGR IRES translation in dicistrovirus-infected cells.

To increase coding capacity, some RNA viruses utilize frameshifting or termination/reinitiation strategies to translate overlapping ORFs [47]. Frameshift events are tightly regulated under different cellular conditions. For example, the extent of programmed –1 frameshifting in human immunodeficiency virus (HIV), which is responsible for generating the precursor of Gag-Pol enzymes, is critical for viral assembly and maturation. Alterations in frameshift efficiency can inhibit viral replication [56], [57]. As a result, the frameshift event has become a strategic antiviral target [58]. Alternatively, it is known that the dynamic levels of polyamines can in part regulate a +1 frameshifting event of the antizyme ORF to control polyamine biosynthesis in cells [59]. The +1 frame protein ORFx of IAPV is detected in virus-infected honey bees suggesting that it has a function during virus infection [25]. However, the function of ORFx has been elusive. Exploring the regulation of +1/0 frame translation may provide clues as to when ORFx is needed during infection. Since translation of both 0 and +1 frames are dependent on the integrity of IAPV IRES, it was important to determine whether the alternate frames are translated either in a fixed ratio or altered during cellular stress or virus infection. In this study, a 20% ratio of +1/0 frame translation was observed using reporter RNA transfections in insect cells, which is similar to the ratio *in vitro* (Figure 4D) [25]. We also find that IAPV IGR IRES +1 frame translation is stimulated to varying extents during cellular stress, similar to that observed monitoring 0 frame translation (Figure 5). Both 0 and +1 frame translation mediated by the IAPV IGR IRES was stimulated to the same extent during CrPV infection in S2 cells, suggesting that translation in both frames are regulated similarly by the IRES (Figure 7). However, these experiments were performed using CrPV infection in S2 cells and it remains to be determined whether the ratio of +1/0 frame translation is regulated under conditions better representing physiological environments such as in IAPV-infected honey bee cells.

IGR IRES activity is stimulated during cellular stress and virus infection. We have also found that sequences adjacent to the IRES contribute to translational activity, which is similar to other findings [19], [25], [31], [33]. Moreover, inclusion of the downstream sequence of the IRES may negatively affect reporter enzymatic activity as we observed with fusion of ORFx with firefly luciferase. The construction of the T2A-containing bicistronic reporter RNA along with the inclusion of adjacent sequences of the IRES should prove useful for expression of exogenous genes using the IGR IRES.

## Supporting Information

Figure S1 **Construction of a T2A-containing 0 frame IRES bicistronic reporter construct.** (A) Schematic of the FLuc reporter gene downstream of the IAPV or CrPV IRES. For clarity, only the PKI domains and a part of the viral structural gene are shown. The ΔPKI mutants that disrupt IGR IRES activities are also shown. (B) Bicistronic reporter constructs with FLuc gene fused in the 0 frame with sORF2 region. T2A-less (left) and T2A-containing (right) bicistronic reporter constructs are shown. A T2A coding sequence is inserted between the sORF2 and FLuc. (C) T2A-less and T2A-containing +1 frame bicistronic constructs were incubated in Sf21 extracts for 120 minutes in the presence of [^35^S]-methionine. The ΔPKI denotes the mutations CC6615-6GG which disrupts PKI basepairing. Note that the T2A-containing reporter constructs resulted in the synthesis of RLuc, 0 frame sORF2-T2A and P-FLuc, and the +1 frame ORFx proteins. (D) Quantitation of radiolabeled FLuc protein products. (E) Quantitation of FLuc and RLuc enzymatic activity from the same translation reactions. (F) *In vitro* transcribed 5′ capped reporter RNAs were incubated in Sf21 lysates for 90 minutes in the presence of [^35^S]-methionine. Quantitations of the FLuc/RLuc ratios are shown normalized to that of the wild-type IAPV IGR IRES. Shown are averages ratios of FLuc/RLuc from at least three independent experiments (± s.d.).
(EPS)

Figure S2 **Sequences adjacent to the IGR IRES contributes to translation.** Bicistronic reporter RNAs containing the core CrPV IGR IRES (nucleotides 6025–6231) or the IRES with the adjacent sequences (‘IRES+’, nucleotides 5974–6372) were transfected into S2 cells. Cells were harvested at 6 hours after transfection and the luciferase activities measured. FLuc, RLuc and the ratio of FLuc/RLuc are normalized to the ‘IRES+’ construct. (C) *In vitro* transcribed IAPV IGR IRES-containing reporter RNAs that were either 5′ capped or uncapped were transfected into S2 cells. Shown are averages from at least three independent experiments (± s.d.).
(EPS)

Figure S3 **IAPV IGR IRES-mediated 0 frame translation in** ***Drosophila*** **Kc167 cells.** Bicistronic reporter RNAs containing the wild-type or mutant (ΔPKI) IAPV IGR IRES were transfected into Kc167 cells. (A) Cells were harvested 6 hours or as indicated after transfection and luciferase activities were measured. Shown are averages from at least three independent experiments (± s.d.). (B) IAPV IGR IRES-mediated 0 frame translation in Kc167 cells treated with DTT.
(EPS)
